# Epigenetically upregulated WIPF1 plays a major role in BRAF V600E-promoted papillary thyroid cancer aggressiveness

**DOI:** 10.18632/oncotarget.13400

**Published:** 2016-11-16

**Authors:** Tao Zhang, Xiaopei Shen, Rengyun Liu, Guangwu Zhu, Justin Bishop, Mingzhao Xing

**Affiliations:** ^1^ Laboratory for Cellular and Molecular Thyroid Research, Division of Endocrinology, Diabetes & Metabolism, Department of Medicine, Johns Hopkins University School of Medicine, Baltimore, MD 21287, USA; ^2^ Department of Pathology, Johns Hopkins University School of Medicine, Baltimore, MD 21287, USA

**Keywords:** WIPF1, thyroid cancer, BRAF V600E, oncogenesis, tumor aggressiveness

## Abstract

How the *BRAF* V600E mutation promotes the pathogenesis and aggressiveness of papillary thyroid cancer (PTC) is not completely understood. Here we explored a novel mechanism involving WASP interacting protein family member 1 (WIPF1). In PTC tumors, compared with the wild-type *BRAF*, *BRAF* V600E was associated with over-expression and hypomethylation of the *WIPF1* gene. In thyroid cancer cell lines with wild-type *BRAF*, *WIPF1* expression was robustly upregulated upon introduced expression of BRAF V600E (*P*=0.03) whereas the opposite was seen upon BRAF knockdown or treatment with BRAF V600E or MEK inhibitors in cells harboring *BRAF* V600E. Methylation of a functionally critical region of the *WIPF1* promoter was decreased by expressing BRAF V600E in cells harboring the wild-type *BRAF* and increased by BRAF knockdown or treatment with BRAF V600E or MEK inhibitors in cells harboring *BRAF* V600E mutation. Under-expression and hypermethylation of *WIPF1* induced by stable BRAF knockdown was reversed by DNA demethylating agent 5′-azadeoxycytidine. Knockdown of WIPF1 robustly inhibited anchorage-independent colony formation, migration, and invasion of thyroid cancer cells and suppressed xenograft thyroid cancer tumor growth and vascular invasion, mimicking the effects of BRAF knockdown. In human PTC tumors, WIPF1 expression was associated with extrathyroidal invasion (*P*=0.01) and lymph node metastasis (*P*=2.64E-05). In summary, BRAF V600E-activated MAP kinase pathway causes hypomethylation and overexpression of *WIPF1*; WIPF1 then functions like an oncoprotein to robustly promote aggressive cellular and tumor behaviors of PTC. This represents a novel mechanism in BRAF V600E-promoted PTC aggressiveness and identifies WIPF1 as a novel therapeutic target for thyroid cancer.

## INTRODUCTION

Follicular cell-derived thyroid cancer is the most common endocrine malignancy with an estimated prevalence of 64,300 for 2016 in the United States [1]. This cancer is histologically classified into papillary thyroid cancer (PTC), follicular thyroid cancer (FTC), poorly differentiated thyroid cancer (PDTC), and anaplastic thyroid cancer (ATC) [2, 3]. Among them, PTC is the most common, currently accounting for close to 90% of all thyroid malignancies [1, 4]. PTC can be further classified into several variants, the most common of which are conventional PTC (CPTC), follicular-variant PTC (FVPTC), and tall-cell PTC (TCPTC) [1, 2, 4, 5], following an aggressiveness order of TCPTC > CPTC >> FVPTC [6].

Of particular interest and importance in understanding the molecular mechanism of thyroid tumorigenesis is the mitogen-activated protein kinase (MAPK) pathway [2]. This pathway is commonly activated by the *BRAF* V600E in human cancers [7], including thyroid cancer [2, 8, 9]. This prominent oncogenic mutation occurs, on average, in approximately 45% of PTC and 24% of ATC [9], which causes a valine-to-glutamic acid substitution at residue 600 of the BRAF protein (V600E), resulting in constitutive activation of the serine/threonine kinase of BRAF and consequent activation of the MAPK pathway. Numerous studies have shown that *BRAF* V600E promotes the aggressiveness of PTC [2, 10, 11]. Multicenter studies demonstrated that *BRAF* V600E mutation was associated with PTC recurrence [12] and PTC-related patient mortality [12, 13]. These clinical behaviors of *BRAF* V600E were recapitulated in transgenic mice in which induced expression of BRAF V600E in the thyroid gland caused PTC that progressed to poorly differentiated invasive carcinomas [14]. Clearly, BRAF V600E plays an important role in the tumorigenesis and aggressiveness of thyroid cancer, yet the underlying molecular mechanisms are not fully understood. One mechanism involves altered expression of genes epigenetically controlled by the BRAF V600E/MAPK pathway [2, 15]. Few such genes, however, have been established and functionally characterized in thyroid cancer pathogenesis and aggressiveness and none is known for any therapeutic targeting potential.

WASP interacting protein family member 1 (WIPF1), a ubiquitously expressed proline-rich multidomain protein, is a binding partner and chaperone of Wiskott-Aldrich syndrome protein (WASP) [16, 17]. Mutations in the WIPF1 binding site of *WASP* or in *WIPF1* itself cause Wiskott-Aldrich syndrome (WAS), which predisposes people to cancer, such as leukemia and lymphoma [18, 19]. Previous studies demonstrated that WIPF1stabilized actin filaments and regulated actin organization and polymerization, which was associated with cell migration and invasion [16, 20–23], apparently in a WASP-independent manner [24, 25]. Interestingly, lower WIPF1 expression was shown to be associated with better prognosis of colorectal cancer, breast cancer, and glioma [19]. WIPF1was also shown to play a role in immune cell functions [25]. These data, taken together, strongly suggest that WIPF1 is a potential cancer gene that may promote cancer aggressiveness.

We previously demonstrated that the MAPK pathway was linked to alterations in promoter methylation of certain genes in thyroid cancer cells, among which was prominently the *WIPF1* gene [26]. Given this finding and the data discussed above, we hypothesized that epigenetically altered WIPF1 might be an important player in mediating BRAF V600E-promoted tumorigenesis and aggressiveness of thyroid cancer. In the present study, we investigated this novel mechanism and the potential of WIPF1 being a novel therapeutic target in thyroid cancer.

## RESULTS

### BRAF V600E up-regulated WIPF1 expression in PTC tumors and thyroid cancer cells

**Figure 1:**
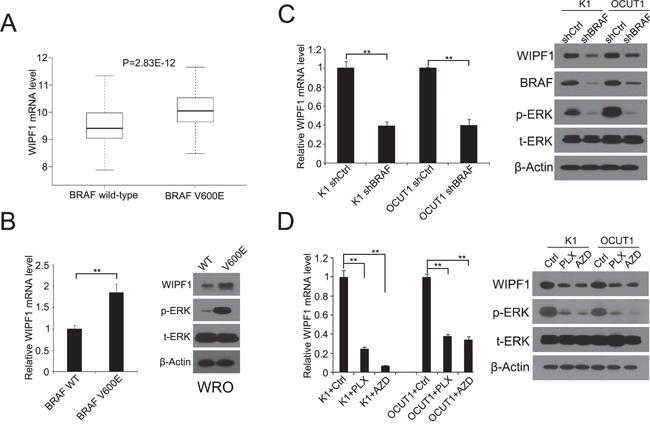
BRAF V600E upregulates WIPF1 expression in thyroid cancer tumor and thyroid cancer cells **A**. mRNA expression levels of WIPF1 were analyzed, RSEM-normalized and log_2_(n+1) transformed in 203 PTC patients without *BRAF* V600E mutation and 287 PTC patients with *BRAF* V600E mutation from the TCGA database (*P*=2.83E-12). **B.** Left panel: Relative mRNA levels of WIPF1 were detected by qRT-PCR in WRO cells stably transfected with BRAF wild-type (WT) or BRAF V600E. Right panel: WIPF1 protein, phosphorylated ERK (p-ERK) and total ERK (t-ERK) were detected by Western blotting in WRO cells stably transfected with BRAF WT or BRAF V600E. **C.** Left panel: Relative mRNA levels of WIPF1 were detected by qRT-PCR in K1 and OCUT1 cells stably transfected with control shRNA (shCtrl) or BRAF shRNA (shBRAF). Right panel: Proteins of WIPF1, BRAF, p-ERK and t-ERK were detected by Western blotting in K1 and OCUT1 cells stably transfected with shCtrl or shBRAF. **D.** Left panel: Relative mRNA levels of WIPF1 were detected by qRT-PCR in K1 and OCUT1 cells treated with the BRAF V600E inhibitor PLX4032 (PLX) at 0.5 μM or the MEK1/2 inhibitor AZD6244 (AZD) at 0.2 μM or DMSO (Ctrl). Right panel: Proteins of WIPF1, p-ERK and t-ERK were detected by Western blotting in K1 and OCUT1 cells treated with 0.5 μM PLX4032 (PLX) or 0.2 μM AZD6244 (AZD) or DMSO (Ctrl). Beta Actin (β-Actin) was used as a loading control in Western blotting analysis. Data were shown as mean±SD of three independent experiments. Statistically significant differences were indicated as *P<0.05; **P<0.01 per two-tailed Student's *t* test.

To examine whether *WIPF1* was functionally a BRAF V600E target gene, we examined the expression of WIPF1in 203 PTC patients without *BRAF* V600E mutation and 287 PTC patients with *BRAF* V600E mutation in the TCGA database [27]. As shown in Figure 1A, the expression of WIPF1 was significantly higher in the *BRAF* V600E-positive group than that in the *BRAF* wild-type group (*P*=2.83E-12). In *in vitro* assay, we confirmed that the mRNA and protein levels of WIPF1 were increased after stably overexpressing BRAF V600E in WRO cell lines which naturally harbored the wild-type BRAF (Figure 1B) [28]. Conversely, we found that the expression of WIPF1 was reduced upon BRAF knockdown in K1 and OCUT1 cells which both harbored *BRAF* V600E (Figure 1C) [28]. These data suggested that the BRAF V600E/MAPK pathway promoted the expression of WIPF1. To further support the role of the BRAF V600E/MAPK pathway in the regulation of *WIPF1*, we demonstrated that WIPF1 expression in K1 and OCUT1 cells was significantly down-regulated after treatment of cells with either the BRAF V600E inhibitor PLX4032 or the MEK inhibitor AZD6244 (Figure 1D).

### BRAF V600E down-regulated methylation of *WIPF1* promoter

**Figure 2:**
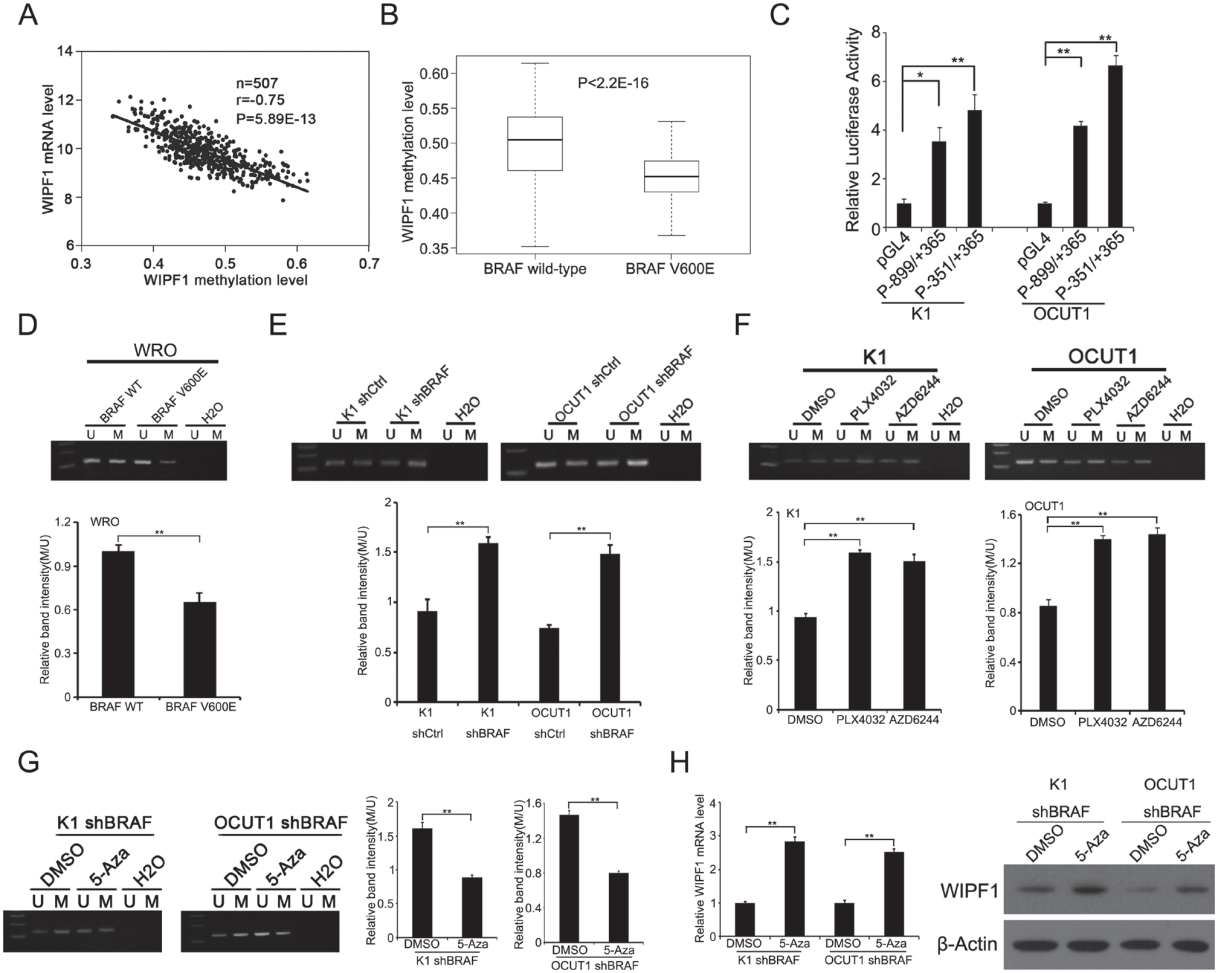
BRAF V600E downregulates methylation of the *WIPF1* promoter **A.** The relationship between WIPF1 mRNA levels and its methylation levels was analyzed in 507 PTC patients from the TCGA database. **B.** The promoter methylation levels of *WIPF1* were examined in 205 PTC patients with *BRAF* wild-type and 287 PTC patients with *BRAF* V600E mutation from the TCGA database (*P*<2.2E-16). **C.** The *WIPF1* promoter activities (P-899/+365 and P-351/+365) were detected in K1 and OCUT1 cells. **D.** The methylation levels of *WIPF1* promoter were examined by methylation-specific PCR (MSP) analysis in WRO cells transfected with BRAF wild-type (WT) or BRAF V600E. U represented the unmethylated and M represented the methylated DNA. H_2_O was used as the negative control for the PCR reaction. The bar graph showed the relative band intensity of the methylation/unmethylation ratio using the Image J software. **E** and **F.** *WIPF1* methylation was examined by MSP in K1 and OCUT1 cells after stable knockdown of BRAF or treatment with the BRAF V600E inhibitor PLX4032 at 0.5 μM or the MEK1/2 inhibitor AZD6244 at 0.2 μM for 72 h. U represented unmethylated and M represented methylated DNA. H_2_O was used as a negative control for the PCR reaction. The bar graph showed the relative band intensity of the methylation/unmethylation ratio using the Image J software. **G** and **H.** *WIPF1* methylation, relative mRNA levels of WIPF1, and protein levels of WIPF1 were examined by MSP, qRT-PCR, and Western blotting, respectively, in K1 and OCUT1 cells with stable BRAF knockdown and with further treatment with control vehicle (DMSO) or 5 μM demethylating agent 5-Aza-2′-deoxycytidine (5-Aza) for 72h. The bar graph in Figure 2G showed the relative band intensity of the methylation/unmethylation ratio using the Image J software. β-Actin was used as a loading control in Western blotting analysis. Data represented mean±SD of three independent experiments. Statistically significant differences were indicated as*P<0.05; **P<0.01 per Student's *t* test.

We previously observed an association of the MAPK pathway with aberrant hypomethylation of a DNA site related to *WIPF1* gene on genome-wide DNA methylation microarray analysis [26]. Here, we investigated directly the role of the BRAF V600E/MAPK pathway in the methylation of the specific gene of *WIPF1* as a mechanism whereby BRAF V600E upregulated the WIPF1 expression. Our analysis of thyroid cancer data in the TCGA database revealed a significant inverse association between the expression of *WIPF1* and its methylation (*r*=−0.75, *P*=5.89E-13, Figure 2A), suggesting that *WIPF1* was epigenetically regulated. We further analyzed the promoter methylation status of *WIPF1* in 205 PTC patients with wild-type *BRAF* and 287 PTC patients with *BRAF* V600E mutation in the TCGA database. We found that *BRAF* V600E mutation was significantly associated with hypomethylation of *WIPF1* compared with the wild-type *BRAF* (*P*<2.2E-16, Figure 2B).

To accurately determine which region in the promoter of the *WIPF1* gene was transcriptively active so methylation of a functionally important region of the *WIPF1* promoter could be specifically studied, we analyzed the promoter of *WIPF1* using the Genomatix software (https://www.genomatix.de/online_help/help_gems/PromoterInspector_help) and constructed the predicted critical 5′-flanking regions of nucleotides -899/+365 and -351/+365 into the report gene vector pGL4.20, respectively. The *WIPF1* promoter activities were examined in K1 and OCUT1 cells using these constructs. We found that the luciferase activities driven by promoter regions -899/+365 and -351/+365 were much higher than the promoterless vector pGL4.20; the activities of -351/+365 were even higher than -899/+365, suggesting that nucleotides -351/+365 represents a functionally important region in the promoter of *WIPF1* (Figure 2C). Moreover, we identified a typical CpG island within the promoter region -351/+365 using the CpG Island Searcher (http://www.ualberta.ca/~stothard/javascript/cpg_islands.html, University of Alberta, Edmonton, Canada). We therefore chose this critical region in the *WIPF1* promoter for methylation analyses using MSP in cells under various conditions. As shown in Figure 2D, methylation of the *WIPF1* promoter was decreased in WRO cells upon stable overexpression of BRAF V600E. Conversely, the *WIPF1* promoter methylation was increased in K1 and OCUT1 cells after shRNA knockdown of BRAF (Figure 2E). Treatment of the cells with the BRAF V600E inhibitor PLX4032 or MEK1/2 inhibitor AZD6244 similarly increased the *WIPF1* promoter methylation (Figure 2F). After treatment with the demethylating agent 5-Aza, K1 and OCUT1 cells in which *WIPF1* was hypermethylated as a result of stable shRNA knockdown of BRAF showed increased mRNA and protein expression of WIPF1 (Figure 2H), accompanied with the corresponding promoter demethylation of the *WIPF1* gene (Figure 2G). As it is well known that promoter methylation usually silences a gene [29], these data suggest that demethylation of the *WIPF1* promoter is a mechanism in the up-regulation of the *WIPF1* gene by the BRAF V600E/MAPK pathway.

### Knockdown of WIPF1 inhibited thyroid cancer cell migration and invasion

**Figure 3:**
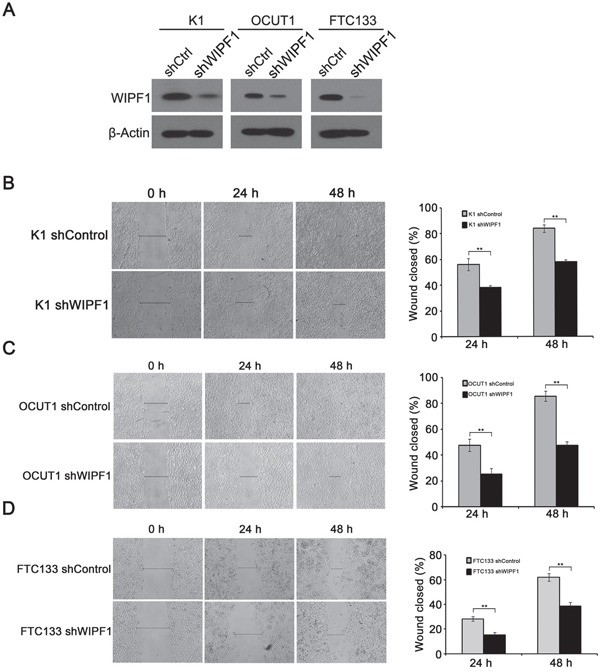
Knockdown of WIPF1 inhibits thyroid cancer cell migration **A.** The protein expression of WIPF1 was examined by Western Blotting in K1, OCUT1 and FTC133 cells after stable transfection with WIPF1 shRNA (shWIPF1) or scrambled control shRNA (shCtrl). **B-D.** Left panel: Stable knockdown of WIPF1 decreased cell motility in K1, OCUT1 and FTC133 cells by wound-healing assay. Wound width was analyzed at 0 h, 24 h and 48 h after wounding. Right panel: The results were presented as the percentage of wound closure as follows: (cell migration distance at the time of measurement/initial wound width) × 100 corresponding to (B-D). The results of each column represented mean±SD of wound closure from three independent experiments. Statistically significant differences were indicated as *P<0.05; **P<0.01 per Student's *t* test.

**Figure 4:**
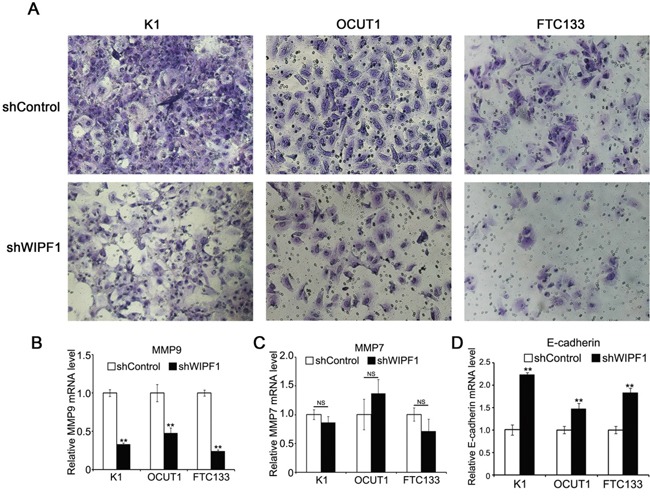
Knockdown of WIPF1 inhibits thyroid cancer cell invasion **A.** K1, OCUT1 and FTC133 cells were stably silenced with WIPF1 shRNA (shWIPF1) followed by the cell invasion assay. Shown were the cells that invaded on the Matrigel matrix-coated polyethylene terephthalate membrane after removal of the non-invasive cells. **B-D.** Relative mRNA levels of MMP9, MMP7 and E-cadherin were determined by qRT-PCR in K1, OCUT1 and FTC133 cells with knockdown of WIPF1 (shWIPF1), respectively. Data were presented as mean ±SD of three independent experiments. Statistically significant differences were indicated as NS, non-significant; *P<0.05; and **P<0.01 per Student's *t* test.

To investigate the role of WIPF1 in thyroid cancer cellular functions, we generated stable K1, OCUT1 and FTC133 cell lines with WIPF1 knockdown using an RNA interference system mediated by a lentivirus vector. As shown in Figure 3A, the expression of WIPF1 was dramatically decreased in these cells. The effect of WIPF1 on the cell migration was examined in these cells using wound healing assay and the migration distance of cells were measured at 0h, 24h, and 48h. The results showed that the cells with depletion of WIPF1 exhibited significantly lower migration compared with the control group without knockdown of WIPF1 in K1, OCUT1 and FTC133 cells (Figures 3B-3D). We also examined the effect of WIPF1 on cell invasion in K1, OCUT1 and FTC133 cells and demonstrated that the number of invasive cells in the WIPF1 knockdown group was dramatically lower than that in the control group without knockdown of WIPF1 (Figure 4A). These data strongly suggest that WIPF1 plays an important role in thyroid cancer cell migration and invasion. As matrix metalloproteinase-7 (MMP7), MMP9 and E-cadherin are known to be involved in cell migration and invasion in some human cancers [30–32], we examined their expression under these conditions. Interestingly, we found that the mRNA level of MMP9 was down-regulated (Figure 4B) and mRNA level of MMP7 did not change (Figure 4C), whereas the mRNA level of E-cadherin was up-regulated (Figure 4D) under the condition of WIPF1 knockdown. These results are consistent with the known role of MMP9 in promoting the cancer cell migration and invasion and the known role of E-cadherin in suppressing cell migration and invasion [30–32].

### Knockdown of WIPF1 suppressed anchorage-independent thyroid cancer cell growth and tumor growth

**Figure 5:**
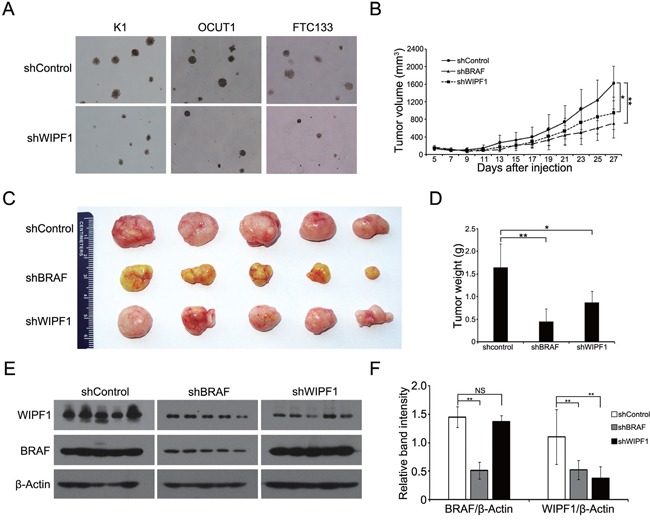
Knockdown of WIPF1 suppresses anchorage-independent cell growth *in vitro* and tumor growth *in vivo* **A.** Representative results of colony formation in K1, OCUT1 and FTC cells after stable knockdown of WIPF1. **B.** Time course of xenograft tumor growth that developed in mice after subcutaneous inoculation with K1 cells transfected with control shRNA (shControl) or BRAF shRNA (shRBAF) (*P*=0.004) or WIPF1 shRNA (shWIPF1) (*P*=0.023). Each time point represented mean±SD of the values obtained from five mice in each group. **C.** Photographs of surgically removed tumors from nude mice at 27 days from the cell inoculation. **D.** Bar graph of average weight of tumors corresponding to (C). **E.** The expression of BRAF and WIPF1 protein was examined by Western blotting in tumor tissues from mice. Different groups of proteins were run on different gels using the same electrophoresis conditions, but their films were exposed and developed together for the same length of time for all the panels shown. **F.** Bar graph presentation of BRAF and WIPF1 protein levels relative to the β-actin levels by calculating the ratios of band intensities of BRAF/β-Actin and WIPF1/β-Actin corresponding to (E). Data were presented as mean ±SD of three independent experiments. Statistically significant differences were indicated: NS, non-significant; *P<0.05; and **P<0.01 per Student's *t* test.

**Figure 6:**
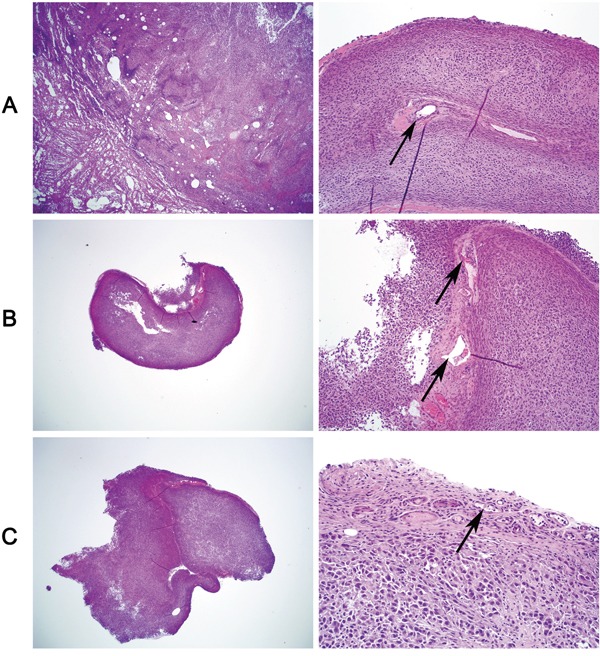
Tumor vascular invasion is suppressed by knockdown of WIPF1 or BRAF in BRAF V600E-bearing thyroid cancer cell-derived tumors Xenograft tumors derived from BRAF V600E-positive K1 cells in nude mice were sectioned for histological H & E staining to examine intravascular invasion. **A.** Vascular invasion of the tumor was seen in the control tumors with intact WIPF1 and BRAF (arrow-pointed). **B** and **C.** No vascular invasion was seen in the tumors with knockdown of WIPF1 (Panel B) or knockdown of BRAF (Panel C). Blood vessels containing blood and fibrin but no tumor cells were seen in the tumors in Panel B (arrow pointed) and small blood vessels containing no tumor cells were seen in the tumors in Panel C (arrow pointed). In each panel, the left portion represented a view at 20X magnification and the right portion represented a view at 200 X magnification of the tumor tissue slide. The figures were representatives of histological staining of the tumors corresponding to Figure 5C.

We next investigated the oncogenic property of WIPF1 in thyroid cancer cells using soft agar colony formation assay. We demonstrated that anchorage-independent cell colony formation of thyroid cancer cells K1, OCUT1 and FTC133 on soft agar was all dramatically decreased with WIPF1 knockdown (Figure 5A). This result suggests an important oncogenic role of WIPF1 as anchorage-independent growth is a classical oncogenic property of cancer cells. We did not observe an effect of WIPF1 on cell proliferation in MTT assay (Supplementary Figures S1A-S1C), suggesting that WIPF1 may not play a major role in cell proliferation or related cell cycle processes. To determine the role of WIPF1 on thyroid cancer tumor growth *in vivo*, K1 cells with stable knockdown of BRAF or WIPF1 were subcutaneously inoculated at the shoulder of mice to produce xenograft tumors. We observed that knockdown of WIPF1 significantly decreased tumor growth both in volume and in the final tumor weight (Figures 5B-5D). BRAF knockdown showed a more profound effect. Correspondingly, on tumor analysis, we confirmed that BRAF protein was dramatically decreased in the BRAF knockdown cells but not in the WIPF1 knockdown cells, while WIPF1 protein was dramatically decreased in cells either with BRAF knockdown or WIPF1 knockdown (Figures 5E and 5F), which was consistent with the above results of reduced WIPF1 expression after BRAF knockdown in the cells. Remarkably, tumor vascular invasion could be observed in control tumors with intact BRAF and WIPF1, while, in contrast, no vascular invasion was observed in the tumors with WIPF1 or BRAF knockdown (Figures 6A-6C).

### Overexpression of *WIPF1* was associated with poor clinicopathological outcomes in PTC in TCGA database

**Figure 7:**
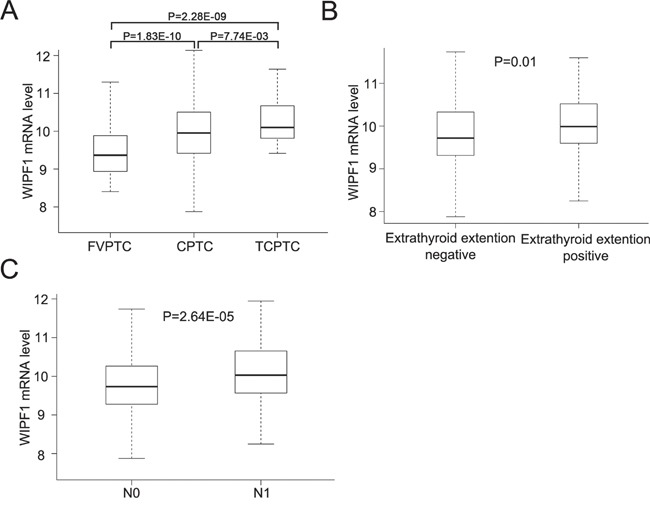
Overexpression of WIPF1 is associated with poor clinicopathological outcomes of PTC in TCGA database **A.** The relationship between WIPF1 mRNA levels and three major subtype of PTC (CPTC, FVPTC and TCPTC) was analyzed from TCGA database. **B** and **C.** The relationship between WIPF1 mRNA levels and extrathyroid invasion or lymph node metastasis was analyzed from TCGA database, respectively. N0: there was no lymph node metastasis. N1: positive for lymph node metastasis.

To further investigate the role of WIPF1 in thyroid cancer, we examined the relationship between WIPF1 expression and clinicopathological outcomes of PTC in the TCGA database. The PTC samples from TCGA database were divided into three pathological subtypes—FVPTC (n=102), CPTC (n=357), and TCPTC (n=35) [5, 6]. A recent study established differential aggressiveness of the three major PTC variants in an order of TCPTC > CPTC >>FVPTC [6]. Remarkably, we found a similar expression order of WIPF1 in these PTC variants. Specifically, WIPF1 expression in FVPTC was significantly lower than that in CPTC and TCPTC (*P*=1.83E-10 and 2.28E-09, respectively); WIPF1 expression in CPTC was significantly lower than that in TCTPC (*P*=7.74E-03, Figure 7A). We also found that WIPF1 expression was significantly associated with tumor extrathyroidal invasion (*P*=0.011, Figure 7B) and lymph node metastasis (*P*=2.64E-05, Figure 7C), suggesting that WIPF1 promotes poor clinicopathological outcomes of thyroid cancer, consistent with an important role of WIPF1 in the aggressiveness of this cancer.

## DISCUSSION

*BRAF* V600E plays a key role in the tumorigenesis and pathogenesis of thyroid cancer [2]. An important phenomenon of this oncogene in thyroid cancer is its widely observed role in the invasion and aggressiveness of PTC [2, 9, 11]. As such, *BRAF* V600E has been recognized as a prognostic genetic molecular marker for poorer prognosis of PTC [33]. Much effort has been devoted to exploring the molecular mechanisms underlying this role of BRAF V600E in recent years, leading to a general consensus that, through aberrantly activating the MAPK pathway, BRAF V600E alters the expression of key genes that are involved in cancer cell activities and tumor pathogenesis [2]. A particularly interesting aspect in this process is the BRAF V600E-coupled alterations in the methylation state of important genes as suggested in our previous genome-wide DNA methylation microarray studies [26], which presumably can cause aberrations in the expression of certain key genes with potentially serious oncogenic consequences. As of today, however, there have been few, if any, genes that are specifically identified to be epigenetically coupled to the BRAF V600E/MAPK pathway and functionally well characterized as playing an important role in the pathogenesis, particularly invasiveness and aggressiveness, of PTC promoted by BRAF V600E. In this context, here we identified and characterized *WIPF1* as such an important gene.

We demonstrated the regulation of the methylation changes in a specific and functionally important region of the promoter of *WIPF1* by the BRAF V600E/MAPK pathway. Specifically, BRAF V600E-activated MAPK pathway promoted demethylation of the *WIPF1* promoter and correspondingly increased the expression of *WIPF1*, which was fully reproduced and confirmed in the analysis of the TCGA data. These results suggest that WIPF1 may have oncogenic properties that confer it an important role in the pathogenesis of thyroid cancer. Indeed, through a variety of *in vitro* studies, we demonstrated a robust role of WIPF1 in the migration and invasion as well as the sustainment of oncogenic properties (i.e., anchorage-independent growth) of thyroid cancer cells. To take a further step to directly show a role of WIPF1 in thyroid tumorigenesis, our *in vivo* xenograft tumor studies demonstrated an important function of WIPF1 in sustaining the growth of thyroid tumor. Interestingly, vascular invasion disappeared in tumors with WIPF1 knockdown as in tumors with BRAF V600E knockdown, providing functionally *in vivo* evidence for a role of WIPF1 in the invasiveness of thyroid cancer. We did not observe a role of WIPF1 in thyroid cancer cell proliferation, suggesting that WIPF1 plays a role in thyroid cancer pathogenesis mainly through its function in promoting cancer cell invasion and tumor aggressiveness. To corroborate this concept is our finding of the role of WIPF1 in the extrathyroidal invasion and lymph node metastasis of human PTC found in the analysis of the TCGA data. This is well consistent with the major role of BRAF V600E in the invasive and metastatic behavior of thyroid cancer [2]. Among the three major PTC variants, TCPTC is known to be the most invasive and aggressive type [5, 6]. It is interesting to note that the expression of WIPF1 was the highest in TCPTC among the three PTC variants in the TCGA data analysis, again consistent with a role of WIPF1 in the development of aggressiveness of thyroid cancer. Thus, when aberrantly up-regulated, WIPF1 possesses a robust oncogenic function in thyroid cancer. This is the first demonstration of such a novel role for WIPF1. These findings are consistent with the report that lower WIPF1 expression was associated with a better prognosis of colorectal cancer, breast cancer, and glioma [19].

Some of the known functions of WIPF1 include stabilization of actin filaments, regulation of actin cytoskeleton organization and polymerization, and interaction with various proteins, such as WASP/N-WASP, HCK and CrkL, which ultimately all affect cell mobility [25, 34]. Moreover, WIPF1 also binds to profilin and cortactin, which are essential for kinetic cellular processes, such as wound healing and cell motility [16, 35]. A recent study reported that WIPF1 played a role in the matrix invasion by breast cancer cells [36]. These functions of WIPF1 may explain its important role in thyroid cancer cell migration and invasion observed in the present study. WIPF1-associated changes in the expression of certain matrix proteins, such as MMP9 and E-cadherin as observed in the present study, that are known to play a role in cancer cell invasion, may also be a contributory mechanism to the role of WIPF1 in the invasiveness of thyroid cancer, although how WIPF1 regulates the expression of these genes needs further investigation.

**Figure 8:**
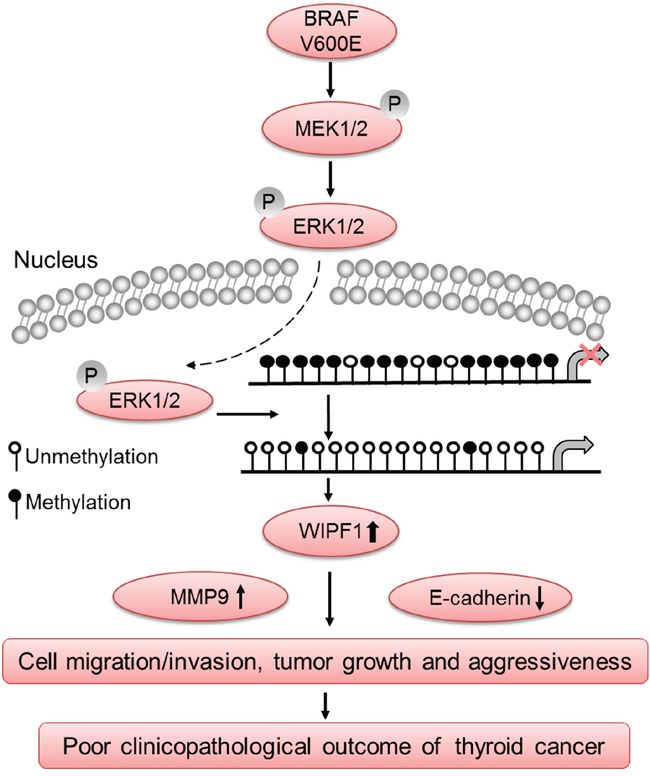
Schematic diagram illustrating the role of WIPF1 in BRAF W600E/MAP kinase pathway-promoted tumorigenesis and aggressiveness of thyroid cancer The BRAF V600E/MAP kinase pathway signaling causes hypomethylation of the *WIPF1* promoter, resulting in over-expression of WIPF1, which in turn promoted thyroid cancer cell migration and invasion and tumor growth, leading to the development of aggressive thyroid cancer. Upregulation of the tumor-promoting molecule MMP9 and down-regulation of tumor suppressor molecule E-cadherin by WIPF1 are involved in this process. This represented a novel mechanism in the widely known BRAF V600E-associagted aggressiveness of thyroid cancer.

BRAF V600E is a well-known driver of the invasiveness and metastasis of PTC, such as extrathyroidal invasion and lymph node metastasis [2, 9, 11]. The present study identified WIPF1 as playing an important mediating role in this function of BRAF V600E. As illustrated in Figure 8, the data in the present study are consistent with a model in which BRAF V600E-activated MAPK pathway causes hypomethylation of the *WIPF1* promoter, hence resulting in over-expression of WIPF1, which in turn promotes the invasiveness and aggressiveness of thyroid cancer.

In summary, the present study identifies the *WIPF1* gene as having novel oncogenic functions and playing an important role in the invasiveness and aggressiveness of thyroid cancer when aberrantly up-regulated by the BRAF V600E/MAPK pathway through its promoter demethylation. This provides a novel mechanism underlying the invasiveness and aggressiveness of PTC promoted by the BRAF V600E and a potential novel therapeutic target for BRAF V600E-harboring thyroid cancer, particularly promising in cases where BRAF V600E inhibitors fail due to drug resistance. Future development of specific treatment strategies, such as the development of WIPF1 inhibitors, can be expected. The findings of the present study have important biological and therapeutic implications for other BRAF V600E-harboring human cancers as well.

## MATERIALS AND METHODS

### Cell lines and reagents

The thyroid cancer cell line K1 was originally from Dr. David Wynford-Thomas (University of Wales College of Medicine, Cardiff, UK); OCUT1 from Dr. Naoyoshi Onoda (Osaka City University Graduate School of Medicine, Osaka, Japan); and WRO-82-1 from Dr. G.J.F. Juillard (University of California-Los Angeles School of Medicine, Los Angeles, CA). These cells were cultured in RPMI Medium 1640 (Gibco, Grand Island, NY, USA) supplemented with 10% fetal bovine serum (FBS, Gibco), 100 U/ml penicillin, and 100 mg/ml streptomycin (Sigma-Aldrich, St. Louis, MO, USA) in 5% CO2 at 37°C. FTC133 was originally from Dr. Georg Brabant (University of Manchester, Manchester, UK) and cultured in Dulbecco's modified Eagle's medium (DMEM)/Ham's F-12 medium (Gibco) supplemented with 10% FBS. The human embryonic kidney cell line 293T (ATCC, Manassas, VA, USA) was maintained in DMEM supplemented with 10% FBS in 5% CO2 at 37°C. All cell lines were routinely authenticated by short tandem repeat analyses and tested for mycoplasma.

The BRAF V600E inhibitor PLX4032 and MEK1/2 inhibitor AZD4244 were purchased from Selleck Chemicals (Houston, TX, USA). Both were dissolved in DMSO at 10 mM stock solution and used to treat cells at 0.5 μM and 0.2 μM, respectively.

### RNA extraction, and quantitative real-time polymerase chain reaction (qRT-PCR)

Total RNA was isolated from cells using TRIzol reagent (Invitrogen, Carlsbad, CA, USA). Two μg of total RNA was converted to cDNA using the SuperScript First-Strand Synthesis System (Invitrogen) on an iCycler Thermal Cycler (Bio-Rad, Philadelphia, PA, USA) following the manufacturer's instructions. QRT-PCR was performed by the Applied Biosystems 7900HT Fast Real-Time PCR System according to the manufacturer's instructions using FastStart Universal SYBR Green Master (Rox) (Roche, Indianapolis, IN, USA). Experiments were conducted in duplicate in three independent assays. Relative transcriptional folds were calculated as 2-ΔΔCt [37]. GAPDH was used as an internal control for normalization. All the primers used are listed in Supplementary Table S1.

### Western blot analysis

Cells were harvested and lysed in RIPA lysis buffer (Santa Cruz Biotechnology, Dallas, TX, USA). Cellular proteins were resolved on denaturing polyacrylamide gels, transferred to the PVDF blotting membrane (GE Healthcare, Germany), and blotted with appropriate primary antibodies. Primary antibodies used included rabbit anti-WIPF1 (Santa Cruz Biotechnology), rabbit anti-ERK1 (Santa Cruz Biotechnology), rabbit anti-phospho-ERK (Cell Signaling Technology, Danvers, MA, USA), mouse anti-BRAF (Santa Cruz Biotechnology), and mouse anti-β-Actin (Santa Cruz Biotechnology). Secondary antibodies included HRP-conjugated anti-rabbit or anti-mouse IgG antibodies from Cell Signaling Technology. Immuno-reactive bands were visualized using the Amersham ECL Prime Western Blotting Detection Reagent (GE Healthcare, UK). All experiments were repeated three times.

### Cell transfection and generation of stable cell lines

Retroviral vectors pBABE-puro (Addgene Inc. Cambridge, MA, USA) containing the full-length wild-type *BRAF* and *BRAF* V600E cDNA were used for stable overexpression of BRAF. Lentiviral pSicoR-PGK-puro vectors (Addgene Inc.) encoding hairpin RNA sequences were used to achieve stable knockdown of BRAF. The pLKO.1-shWIPF1 (Sigma) was used to achieve knockdown of WIPF1. To generate lentiviral particles, human embryonic kidney 293T cells were co-transfected with the viral vector and compatible packaging plasmid mixture (psPAX2 and pMD2.G, Addgene Inc.) using Lipofectamine 3000 (Invitrogen) following the manufacturer's instructions. K1, OCUT1, FTC133 and WRO cells were exposed to virus-containing supernatant for 16 hours in the presence of 8 μg/mL polybrene (Sigma). After 7-10 days of selection with puromycin (Sigma), cells were serum-starved (0.5% FBS) and harvested 24 hours later in RIPA lysis buffer (Santa Cruz Biotechnology). The expression of BRAF, p-ERK, and WIPF1 was examined by Western blotting.

### Luciferase reporter gene assay

The 5′-flanking region (-899/+365 and -351/+365) of *WIPF1* was amplified by PCR from the genomic DNA of K1 cell using specific primers and was cloned into the upstream of the pGL4.20 (Promega) via KpnI and Hind III sites, respectively. All primers are listed in Supplementary Table S1. Luciferase reporter gene assay was performed in K1 and OCUT1 cells using the Dual-Luciferase Reporter Assay System (Promega) according to the manufacturer's instructions. Cells were transferred into 24-well plates at 3 ×10^4^ cells per well. After 24 h, cells were transiently co-transfected with 0.1 μg/well of pRL-TK plasmid (Promega) containing the Renilla luciferase gene used for internal normalization, and various constructs containing different lengths of the WIPF1 5′-flanking region or promoterless vector pGL4.20. After 48h, the luciferase activities were measured as previously described [38]. All experiments were performed at least three times.

### Methylation-specific PCR (MSP)

Genomic DNA was extracted from cells using QIAamp DNA Mini Kit (QIAGEN, Hilden, Germany). One μg genomic DNA was modified with sodium bisulfite using an EZ DNA Methylation-Gold™ Kit according to the manufacturer's instructions (Zymo Research, Irvine, CA). MSP primers were designed using the Methyl Primer Express Software version 1.0 (Life Technologies, Grand Island, NY) and MSP was performed as described previously [39]. The primers used were presented in Supplementary Table S1.

### Wound healing assay

Cells were seeded in 6-well plates and grew up to about 90% confluence before wounding with a 200 μl plastic tip across the monolayer cells. The debris was removed by washing three times with PBS, and cells were then cultured with fresh complete medium. The images were captured at 0, 24, and 48 h after wounding (Nikon Eclipse Ti-U, Tokyo, Japan). The migration distance was calculated using the following formula: migration distance (%) = (wound width at the beginning-wound width at each time point)/wound width at the beginning. Each experiment was performed in triplicate.

### Transwell invasion assay

The cell invasion assay was performed according to the instructions of the assay kit's manufacturer. Briefly, Matrigel invasion chambers with 6.5-mm diameter and 8-μm pores coated with Matrigel matrix (BD BioCoat™ Matrigel™ Invasion Chamber, BD Biosciences) were used in the cell invasion assay. After serum starvation for 8 h, 5×10^4^ of K1, OCUT1 and FTC133 cells stably transfected with the negative control pLKO.1 containing scramble shRNA and lentiviral shRNA plasmid shWIPF1 were re-suspended with 250 μl of serum-free 1640 or DMEM/F12. Cell suspensions were added into the upper chamber of the Transwell insert and 750 μl of 1640 or DMEM/F12 media containing 10% FBS was added to the lower compartment of each well. After 22h of incubation at 37°C with 5% CO2, the noninvasive cells in the upper chamber were removed and the invaded cells on the lower side of the membrane were fixed with 100% methanol and stained with 0.5% crystal violet in 2% ethanol. The invasive cells were photographed under a microscope (Nikon Eclipse Ti-U).

### Analysis of cell proliferation

Cells were seeded onto 96-well plates (1000 cells/well) and 3-(4,5-dimethylthiazol-2-yl)-2,5-diphenyltetrazolium bromide (MTT) assay was used to assess cell proliferation daily from the first day to the fourth day as described previously [40].

### Colony formation assay

For soft-agar colony formation assay, 2×10^3^ cells for K1 and FTC133 or 1×10^4^ cells for OCUT1 were plated into 6-well plates with a bottom layer of 0.6% agar and a top layer of 0.3% agar. Following the hardening of soft agar, plates were incubated at 37°C in a humidified 5% CO2 incubator. After two weeks of incubation, colonies were captured under a microscope (Nikon Eclipse Ti-U).

### Animal xenograft tumor assay

Nude mice were housed and treated according to guidelines established by the National Institutes of Health for the Care and Use of Laboratory Animals. Xenograft tumors were produced by inoculating thyroid cancer cells subcutaneously in nude mice following our standard protocols. Briefly, K1 cells with stable transfection of control shRNA, BRAF shRNA or WIPF1 shRNA were harvested and re-suspended at 2×10^7^ per ml with sterile PBS. Three groups of 4-week-old female BALB/c athymic nude mice (Harlan Sprague Dawley, Indianapolis) (each group, n=5) were subcutaneously injected at the shoulder with 0.2 ml of cell suspensions. Tumor growth was measured after 5 days from injection and then every 2 days. Tumor volume (V) was monitored by measuring the length (L) and width (W) with calipers and calculated with the formula (L × W^2^) × 0.5. After 27 days, all tumor-bearing mice were sacrificed, and the tumors were excised and weighted. Tumor tissue slides were sectioned for H&E staining and vascular examination. Proteins were extracted from tumor tissues using the RIPA lysis buffer (Santa Cruz Biotechnology) and the protein expression of BRAF and WIPF1 were examined by Western blotting.

### The cancer genome atlas (TCGA) data analysis

DNA methylation, RNAseq, and clinical data were obtained from the TCGA Genome Data Analysis Center Firehose website (http://firebrowse.org/). All data were downloaded from the November 1, 2015 standard data. For the methylation data, only probes located in transcriptional regulatory regions, including TSS1500, TSS200, 5′-UTR, enhancer and 1st exon regions were used to calculate the average methylation values of the promoter region of *WIPF1*. For RNAseq data, expression levels were RSEM-normalized and log_2_(n+1) transformed. *BRAF* V600E somatic mutation were calculated by MuTect with the raw exon sequencing data in 496 patients from TCGA [41].

### Statistical analysis

Each experiment was repeated at least three times. Statistical significance was assessed by comparing mean values (± SD) using a two-sided Student's t test for independent groups and was assumed for *P*<0.05 (*) and *P*<0.01(**). Pearson correlation coefficient was calculated to evaluate the relationship between WIPF1 expression and *WIPF1* methylation levels in PTC from the TCGA database.

## SUPPLEMENTARY FIGURE AND TABLE



## References

[1] Howlader N, Noone AM, Krapcho M, Miller D, Bishop K, Altekruse SF, Kosary CL, Yu M, Ruhl J, Tatalovich Z, Mariotto A, Lewis DR, Chen HS (2016). SEER Cancer Statistics Review, 1975-2013.

[2] Xing M (2013). Molecular pathogenesis and mechanisms of thyroid cancer. Nat Rev Cancer.

[3] Tuttle RM, Ball DW, Byrd D, Dilawari RA, Doherty GM, Duh QY, Ehya H, Farrar WB, Haddad RI, Kandeel F, Kloos RT, Kopp P, Lamonica DM (2010). Thyroid carcinoma. J Natl Compr Canc Netw.

[4] Mao Y, Xing M (2016). Recent incidences and differential trends of thyroid cancer in the United States. Endocr Relat Cancer.

[5] Lam AK, Lo CY, Lam KS (2005). Papillary carcinoma of thyroid: A 30-yr clinicopathological review of the histological variants. Endocr Pathol.

[6] Shi X, Liu R, Basolo F, Giannini R, Shen X, Teng D, Guan H, Shan Z, Teng W, Musholt TJ, Al-Kuraya K, Fugazzola L, Colombo C (2016). Differential Clinicopathological Risk and Prognosis of Major Papillary Thyroid Cancer Variants. J Clin Endocrinol Metab.

[7] Davies H, Bignell GR, Cox C, Stephens P, Edkins S, Clegg S, Teague J, Woffendin H, Garnett MJ, Bottomley W, Davis N, Dicks E, Ewing R (2002). Mutations of the BRAF gene in human cancer. Nature.

[8] Cohen Y, Xing M, Mambo E, Guo Z, Wu G, Trink B, Beller U, Westra WH, Ladenson PW, Sidransky D (2003). BRAF mutation in papillary thyroid carcinoma. J Natl Cancer Inst.

[9] Xing M (2005). BRAF mutation in thyroid cancer. Endocr Relat Cancer.

[10] Xing M, Westra WH, Tufano RP, Cohen Y, Rosenbaum E, Rhoden KJ, Carson KA, Vasko V, Larin A, Tallini G, Tolaney S, Holt EH, Hui P (2005). BRAF mutation predicts a poorer clinical prognosis for papillary thyroid cancer. J Clin Endocrinol Metab.

[11] Xing M (2007). BRAF mutation in papillary thyroid cancer: pathogenic role, molecular bases, and clinical implications. Endocr Rev.

[12] Xing M, Alzahrani AS, Carson KA, Shong YK, Kim TY, Viola D, Elisei R, Bendlova B, Yip L, Mian C, Vianello F, Tuttle RM, Robenshtok E (2015). Association Between BRAF V600E Mutation and Recurrence of Papillary Thyroid Cancer. J Clin Oncol.

[13] Xing M, Alzahrani AS, Carson KA, Viola D, Elisei R, Bendlova B, Yip L, Mian C, Vianello F, Tuttle RM, Robenshtok E, Fagin JA, Puxeddu E (2013). Association between BRAF V600E mutation and mortality in patients with papillary thyroid cancer. JAMA.

[14] Knauf JA, Ma X, Smith EP, Zhang L, Mitsutake N, Liao XH, Refetoff S, Nikiforov YE, Fagin JA (2005). Targeted expression of BRAFV600E in thyroid cells of transgenic mice results in papillary thyroid cancers that undergo dedifferentiation. Cancer Res.

[15] Xing M (2007). Gene methylation in thyroid tumorigenesis. Endocrinology.

[16] Ramesh N, Antón IM, Hartwig JH, Geha RS (1997). WIP, a protein associated with wiskott-aldrich syndrome protein, induces actin polymerization and redistribution in lymphoid cells. Proc Natl Acad Sci U S A.

[17] de la Fuente MA, Sasahara Y, Calamito M, Antón IM, Elkhal A, Gallego MD, Suresh K, Siminovitch K, Ochs HD, Anderson KC, Rosen FS, Geha RS, Ramesh N (2007). WIP is a chaperone for Wiskott-Aldrich syndrome protein (WASP). Proc Natl Acad Sci U S A.

[18] Lanzi G, Moratto D, Vairo D, Masneri S, Delmonte O, Paganini T, Parolini S, Tabellini G, Mazza C, Savoldi G, Montin D, Martino S, Tovo P (2012). A novel primary human immunodeficiency due to deficiency in the WASP-interacting protein WIP. J Exp Med.

[19] Staub E, Groene J, Heinze M, Mennerich D, Roepcke S, Klaman I, Hinzmann B, Castanos-Velez E, Pilarsky C, Mann B, Brummendorf T, Weber B, Buhr HJ (2009). An expression module of WIPF1-coexpressed genes identifies patients with favorable prognosis in three tumor types. J Mol Med (Berl).

[20] Sasahara Y, Rachid R, Byrne MJ, de la Fuente MA, Abraham RT, Ramesh N, Geha RS (2002). Mechanism of recruitment of WASP to the immunological synapse and of its activation following TCR ligation. Mol Cell.

[21] Antón IM, Lu W, Mayer BJ, Ramesh N, Geha RS (1998). The Wiskott-Aldrich syndrome protein-interacting protein (WIP) binds to the adaptor protein Nck. J Biol Chem.

[22] Donnelly SK, Weisswange I, Zettl M, Way M (2013). WIP provides an essential link between Nck and N-WASP during Arp2/3-dependent actin polymerization. Curr Biol.

[23] Scott MP, Zappacosta F, Kim EY, Annan RS, Miller WT (2002). Identification of novel SH3 domain ligands for the Src family kinase Hck. Wiskott-Aldrich syndrome protein (WASP), WASP-interacting protein (WIP), and ELMO1. J Biol Chem.

[24] Antón IM, de la Fuente MA, Sims TN, Freeman S, Ramesh N, Hartwig JH, Dustin ML, Geha RS (2002). WIP deficiency reveals a differential role for WIP and the actin cytoskeleton in T and B cell activation. Immunity.

[25] Fried S, Matalon O, Noy E, Barda-Saad M (2014). WIP: more than a WASp-interacting protein. J Leukoc Biol.

[26] Hou P, Liu D, Xing M (2011). Genome-wide alterations in gene methylation by the BRAF V600E mutation in papillary thyroid cancer cells. Endocr Relat Cancer.

[27] Cancer Genome Atlas Research Network (2014). Integrated genomic characterization of papillary thyroid carcinoma. Cell.

[28] Liu D, Yang C, Bojdani E, Murugan AK, Xing M (2013). Identification of RASAL1 as a major tumor suppressor gene in thyroid cancer. J Natl Cancer Inst.

[29] Robertson KD (2005). DNA methylation and human disease. Nat Rev Genet.

[30] Jeanes A, Gottardi CJ, Yap AS (2008). Cadherins and cancer: how does cadherin dysfunction promote tumor progression?. Oncogene.

[31] Shiomi T, Okada Y (2003). MT1-MMP and MMP-7 in invasion and metastasis of human cancers. Cancer Metastasis Rev.

[32] Yu Q, Stamenkovic I (2000). Cell surface-localized matrix metalloproteinase-9 proteolytically activates TGF-beta and promotes tumor invasion and angiogenesis. Genes Dev.

[33] Xing M, Haugen BR, Schlumberger M (2013). Progress in molecular-based management of differentiated thyroid cancer. Lancet.

[34] Calle Y, Antón IM, Thrasher AJ, Jones GE (2008). WASP and WIP regulate podosomes in migrating leukocytes. J Microsc.

[35] Banon-Rodriguez I, Monypenny J, Ragazzini C, Franco A, Calle Y, Jones GE, Antón IM (2011). The cortactin-binding domain of WIP is essential for podosome formation and extracellular matrix degradation by murine dendritic cells. Eur J Cell Biol.

[36] García E, Machesky LM, Jones GE, Antón IM (2014). WIP is necessary for matrix invasion by breast cancer cells. Eur J Cell Biol.

[37] Schmittgen TD, Livak KJ (2008). Analyzing real-time PCR data by the comparative C(T) method. Nat Protoc.

[38] You X, Liu F, Zhang T, Lv N, Liu Q, Shan C, Du Y, Kong G, Wang T, Ye L, Zhang X (2014). Hepatitis B virus X protein upregulates Lin28A/Lin28B through Sp-1/c-Myc to enhance the proliferation of hepatoma cells. Oncogene.

[39] Xing M, Usadel H, Cohen Y, Tokumaru Y, Guo Z, Westra WB, Tong BC, Tallini G, Udelsman R, Califano JA, Ladenson PW, Sidransky D (2003). Methylation of the thyroid-stimulating hormone receptor gene in epithelial thyroid tumors: a marker of malignancy and a cause of gene silencing. Cancer Res.

[40] Liu D, Hou P, Liu Z, Wu G, Xing M (2009). Genetic Alterations in the Phosphoinositide 3-Kinase/Akt Signaling Pathway Confer Sensitivity of Thyroid Cancer Cells to Therapeutic Targeting of Akt and Mammalian Target of Rapamycin. Cancer Res.

[41] Cibulskis K, Lawrence MS, Carter SL, Sivachenko A, Jaffe D, Sougnez C, Gabriel S, Meyerson M, Lander ES, Getz G (2013). Sensitive detection of somatic point mutations in impure and heterogeneous cancer samples. Nat Biotechnol.

