# Supplementary material for: Epigenetically upregulated WIPF1 plays a major role in BRAF V600E-promoted papillary thyroid cancer aggressiveness
Source: Oncotarget. 2016 Nov 16;8(1):900–14. doi: 10.18632/oncotarget.13400 (PMC5352205; doi:10.18632/oncotarget.13400)
Supplement: Supplementary file 1 [file oncotarget-08-900-s001.pdf]

Epigenetically upregulated WIPF1 plays a major role in BRAF V600E-promoted papillary thyroid cancer aggressiveness

SUPPLEMENTARY FIGURE AND TABLE

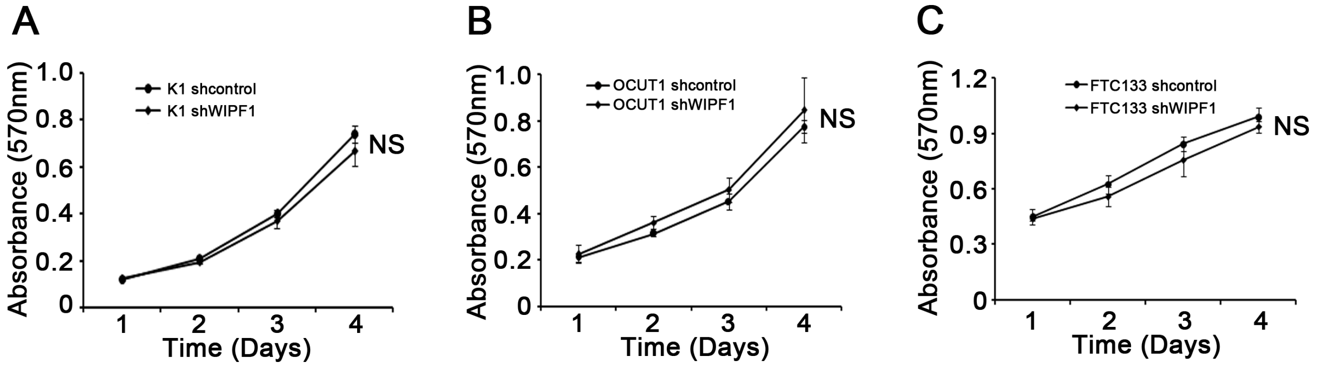

Supplementary Figure S1: Knockdown of WIPF1 doesn't affect cell proliferation. A-C. The cell proliferation was examined by MTT in K1, OCUT1 and FTC133 cells with stable knockdown of WIPF1 at 1 day, 2 day, 3 day and 4 day. Statistically significant differences were indicated: NS, non-significant;**P*<0.05; ***P*<0.01; Student's *t* test.

Supplementary Table S1: List of primers used in this paper

Gene	Primer	Sequence(5′-3′)
Primers for qRT-PCR		
WIPF1	forward	AGTTCCAGCGGCAATGACG
	reverse	GATGTGCTGCCGTTTCTGC
MMP7	forward	GATGAGGATGAACGCTGGAC
	reverse	AGGATCAGAGGAATGTCCCA
MMP9	forward	TTGACAGCGACAAGAAGTGG
	reverse	GCCATTCACGTCGTCCTTAT
E-cadherin	forward	GAAGGTGACAGAGCCTCTGGAT
	reverse	GATCGGTTACCGTGATCAAAATC
GAPDH	forward	TGCACCACCAACTG CTTAGC
	reverse	GGCATGGACTGTGGTCATGAG
Primers for WIPF1 promoter		
P-899/+365	forward	CGATGGTTATTACACTGAGC
	reverse	CATCAGCGGGCAGCAGAAC
P-351/+365	forward	CAAGAAGGTGGAGAAGATTC
	reverse	CATCAGCGGGCAGCAGAAC
Primers for MSP		
Unmethylated	forward	TAAATTTTTGTTTTTTTGTTTTGG
	reverse	AAAACTCTACACCTACAACTTCAAC
Methylated	forward	GTAAATTTTTGTTTTTTTGTTTCGG
	reverse	ATAAAACTCTACGCCTACAACTTCG
